# Individualized strategy of minimally invasive cardiac surgery in congenital cardiac septal defects

**DOI:** 10.1186/s13019-022-01753-6

**Published:** 2022-01-15

**Authors:** Jiaquan Zhu, Yunjiao Zhang, Chunrong Bao, Fangbao Ding, Ju Mei

**Affiliations:** Department of Cardiothoracic Surgery, Xinhua Hospital, Shanghai Jiaotong University School of Medicine, 1665 Kongjiang Road, Shanghai, 200092 China

**Keywords:** Congenital heart disease, Minimally invasive cardiac surgery, Intracardiac septal defect

## Abstract

**Background:**

Intracardiac septal defect is repaired using median sternotomy in most centers; however, there are several reports using minimally invasive surgery in both children and adults. This study summarized our strategy of minimally invasive therapy using various lateral mini-thoracotomies in patients with congenital septal defect.

**Methods:**

In this study, 472 patients who underwent minimally invasive repair of intracardiac septal defects (atrial septal defect, (ASD), ventricular septal defect, (VSD), and atrioventricular septal defect, (AVSD)) from January 2012 to June 2020 were retrospectively reviewed. Those who underwent device closure were excluded. The minimally invasive strategy included three groups: the right sub-axillary vertical incision (RSAVI) group (N = 335, including192 ASDs, 135 VSDs and 8 AVSDs); the right anterolateral thoracotomy (RALT) group (N = 132, including 77 ASDs, 51 VSDs and 4 AVSDs); and the left anterolateral thoracotomy (LALT) group (N = 5, all subpulmonary VSDs).

**Results:**

Concomitant surgeries included nine cases of right ventricular outflow tract obstruction relief, nine cases of mitral repairs and 37 cases of tricuspid repairs. There was one transition from thoracotomy to sternotomy. Three patients required second pump run for residual lesions (two residual VSD shunts and one mitral regurgitation). The age and body weight of the RSAVI group were significantly lower than those of the RALT and LALT groups (all *P* < 0.01). No postoperative death was observed. Postoperative complications included one case of chest exploration for bleeding, one case of reoperation due to patch dehiscence during the same admission, one case of transient neural dysfunction, three cases of diaphragmatic paresis and 13 cases of atelectasis. The median stay in the intensive care unit was two days, while the median postoperative hospitalization duration was six days. The echocardiography results before discharge indicated no significant residual lesions. No reoperation, no new onset of chest deformities and no sclerosis were observed during the follow-up.

**Conclusions:**

Intracardiac septal defects can be safely and effectively repaired by minimally invasive surgery with good cosmetic results. RSAVI is suitable in infants and children, while RALT is more commonly used in adolescents and adults. LALT is an alternative incision to repair subpulmonary VSD.

## Background

Congenital cardiac septal defects include atrial septal defect (ASD), ventricular septal defect (VSD), atrioventricular septal defect (AVSD) and their combinations. They account for half of all congenital heart diseases. Surgical repair of such defects using median sternotomy was developed several decades ago and is widely used as a standard approach in many cardiac centers. The surgical results are excellent, and the mortality is approaching to 0% today. However, the median scar on the chest reminds the patient of the surgical history which even leads to emotional issues and psychological diseases. In the 1990s, device closure for ASD or VSD via either percutaneous or mini-thoracotomy was successfully applied in appropriate candidates and it has advantages of less trauma, fast postoperative recovery, and cosmetic effects [1, 2]. Nevertheless, several patients with ASD or VSD cannot be treated with device closure because of inappropriate anatomical features. These patients still require surgical repair with cardiopulmonary bypass.

Minimally invasive cardiac surgery (MICS) became popular in adult heart surgery, and the principle is to minimize trauma maintaining balance between safety and efficacy. Multiple heart centers have reported their experiences of MICS in pediatric and adult congenital heart surgery worldwide. These techniques include repair from right anterolateral mini-thoracotomy (RALT), right oblique subaxillary incision, right vertical subaxillary incision, right posterior mini-thoracotomy, and partial sternotomy [3–12]. These techniques not only had their own advantages but also had limitations. Right sub-axillary vertical incision (RSAVI) was widely used; however, it cannot provide sufficient exposure in subpulmonary VSD and was not convenient in adolescent and adult patients. In our institution, individualized MICS was used in repairing congenital heart defects based on anatomical features and patient characteristics. Hereby, our experience of the last decade was summarized.

## Methods

### Patient selection

This study was approved by the ethic committee of our institution, and consent from the patients was waived. Patients who underwent repair of cardiac septal defects from January 2012 to June 2020 in Shanghai Xinhua Hospital were retrospectively reviewed. The patients were screened for their surgical techniques. Those who underwent minimally invasive repair with cardiopulmonary bypass were included for analysis, whereas those who underwent repair through sternotomy were excluded. Those who underwent device closure either percutaneously or per mini-thoracotomy were excluded as well. The approaches of MICS include three kinds of incisions. In group 1, RSAVI was used (RSAVI group, N = 335); in group 2, RALT was used (RALT group, N = 132), and in group 3, left anterolateral thoracotomy was used (LALT group, N = 5).

### Surgical techniques

#### RSAVI group

**Fig. 1:**
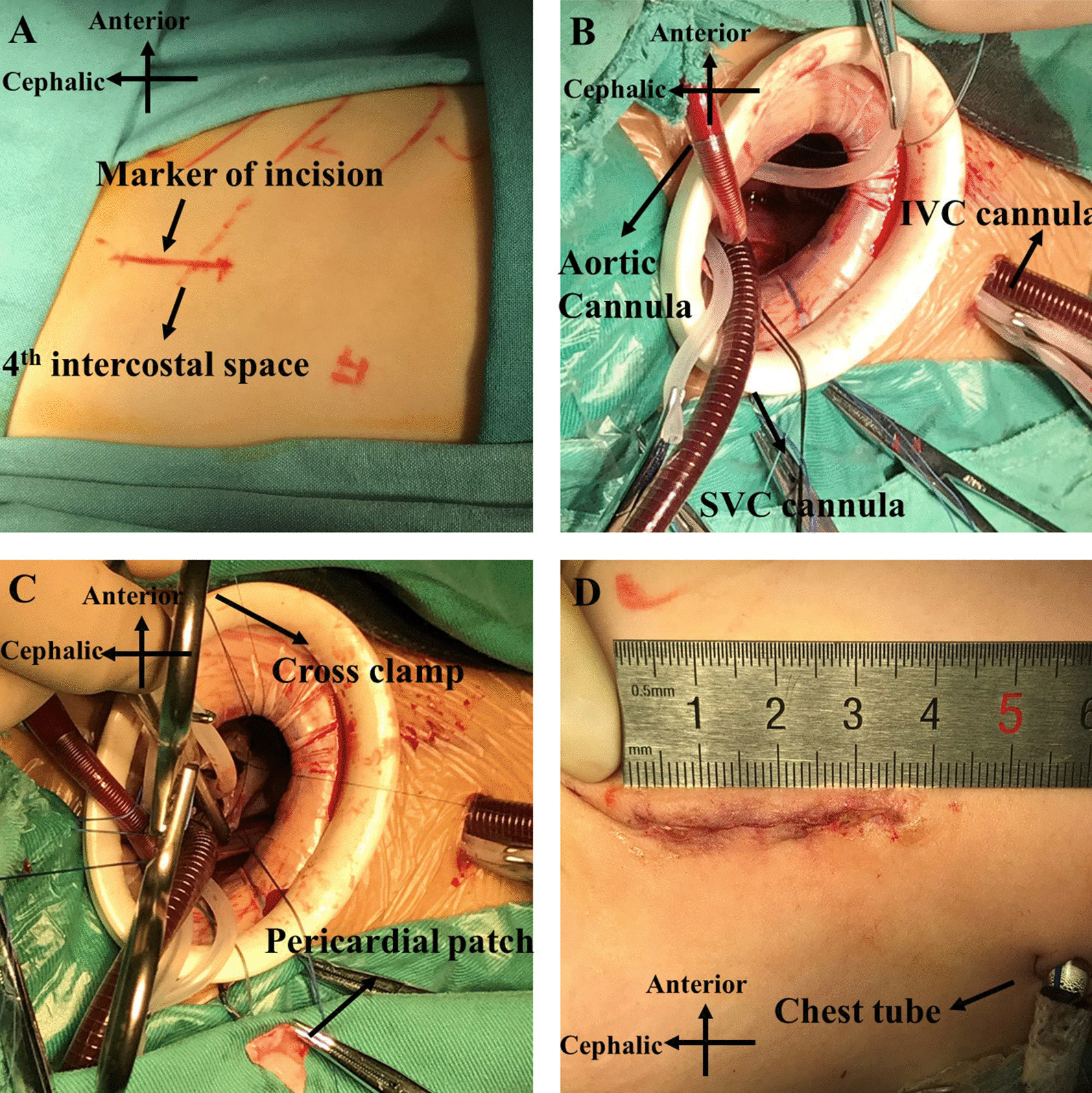
A surgeon’s view of minimally invasive cardiac surgery from a right subaxillary vertical incision. **A** Markers of incision and fourth intercostal space. **B** Setup of the arterial cannula and vena cava cannulas. **C** repair of the cardiac defect with a piece of the pericardium. **D** Skin incision and chest tube at the end of the operation

This technique was mainly used in infants and children. The technique is similar to a previously reported method [13]. The patient was intubated using a single-lumen tube after general anesthesia; then, the patient was placed laterally with the right side up. The right arm was placed cranially or suspended on a head brace. An approximately 4-cm vertical incision was made across the fourth intercostal space. The subcutaneous tissue was dissected with caution to protect the long thoracic nerve and its associated blood vessels. The chest was entered through the fourth intercostal space. A rigid or soft tissue spreader was placed to gain better exposure. A wet sponge was used to protect the right lung while retracting. Then, the right half thymus was resected. A longitudinal pericardial incision was made approximately 1.5 cm anterior to the right phrenic nerve, and several retraction stitches were placed to help expose the heart. After heparinization, the aortic and bicaval cannulations were placed through the incision. Then, an additional 1 cm incision was made in the sixth intercostal space, and the inferior vena cava (IVC) cannula was pulled out from this hole which was used to place a chest tube at the end of the operation (Fig. 1). A cardioplegia needle was placed at the aortic root. After an aortic cross clamp was placed, del Nido cardioplegia was given and the heart was arrested. The right atrium was open, and intracardiac defects were repaired after that. The repair technique was almost the same as that from sternotomy. In some cases, especially in patients with VSD, the distance from the incision to the defect was too deep to obtain adequate exposure. A wet ice-cold sponge was placed behind the heart in the pericardial cavity, thereby the heart was elevated and closer to the surgeon.

#### RALT group

**Fig. 2:**
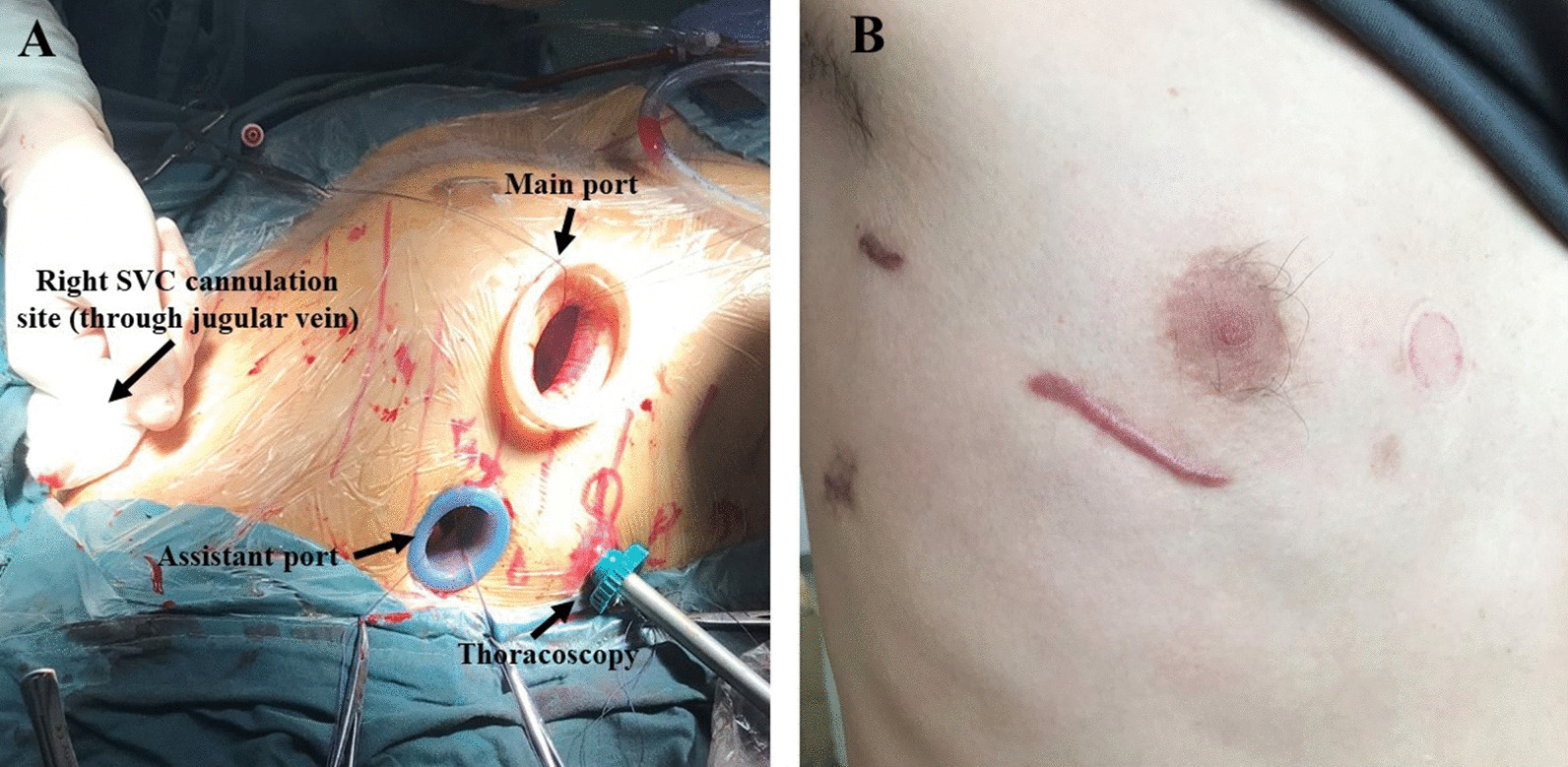
Diagram of right anterolateral incision with thoracoscopy assistance. **A** Setup of the three ports. The main and assistant ports were in the fourth intercostal space, while the thoracoscopy port was in the fifth or sixth intercostal space. A left ventricular vent and Chitwood cross-clamp are placed through the assistant port. A superior vena cava cannula is placed through the right jugular vein. **B** Skin incision three months after the operation

This technique is used mainly in older children, adolescents, and adults. The patient was placed in the supine position; then, the right side was elevated approximately 30 degrees. The patient was intubated using a double-lumen tube if feasible. A right anterolateral small incision was made at the fourth intercostal space. In female patients, this incision was lower at the margin of breast tissue. The chest cavity was entered through the fourth intercostal space. In our early experience, a rigid spreader was inserted to expose the heart. The pericardium was opened 2 cm anterior to the right phrenic nerve. A vertical right groin incision was made, then the femoral artery and vein were mobilized for peripheral cannulation to establish cardiopulmonary bypass. In our earlier series, a superior vena cava (SVC) canula and a cardioplegia needle were placed through the incision, and the aorta was cross-clamped; then, the intracardiac defects were repaired. Recently, modifications were made to further minimize injury. The incision was made less than 5 cm. A soft tissue retractor was used, and a rigid retractor was abandoned. Thoracoscopy was used for better exposure (Fig. 2). An SVC cannula was placed through the internal jugular vein using the Seldinger technique. A Chitwood aortic cross clamp was placed through the third or fourth intercostal space. Therefore, the incision was spared for defect repair only.

#### LALT group

**Fig. 3:**
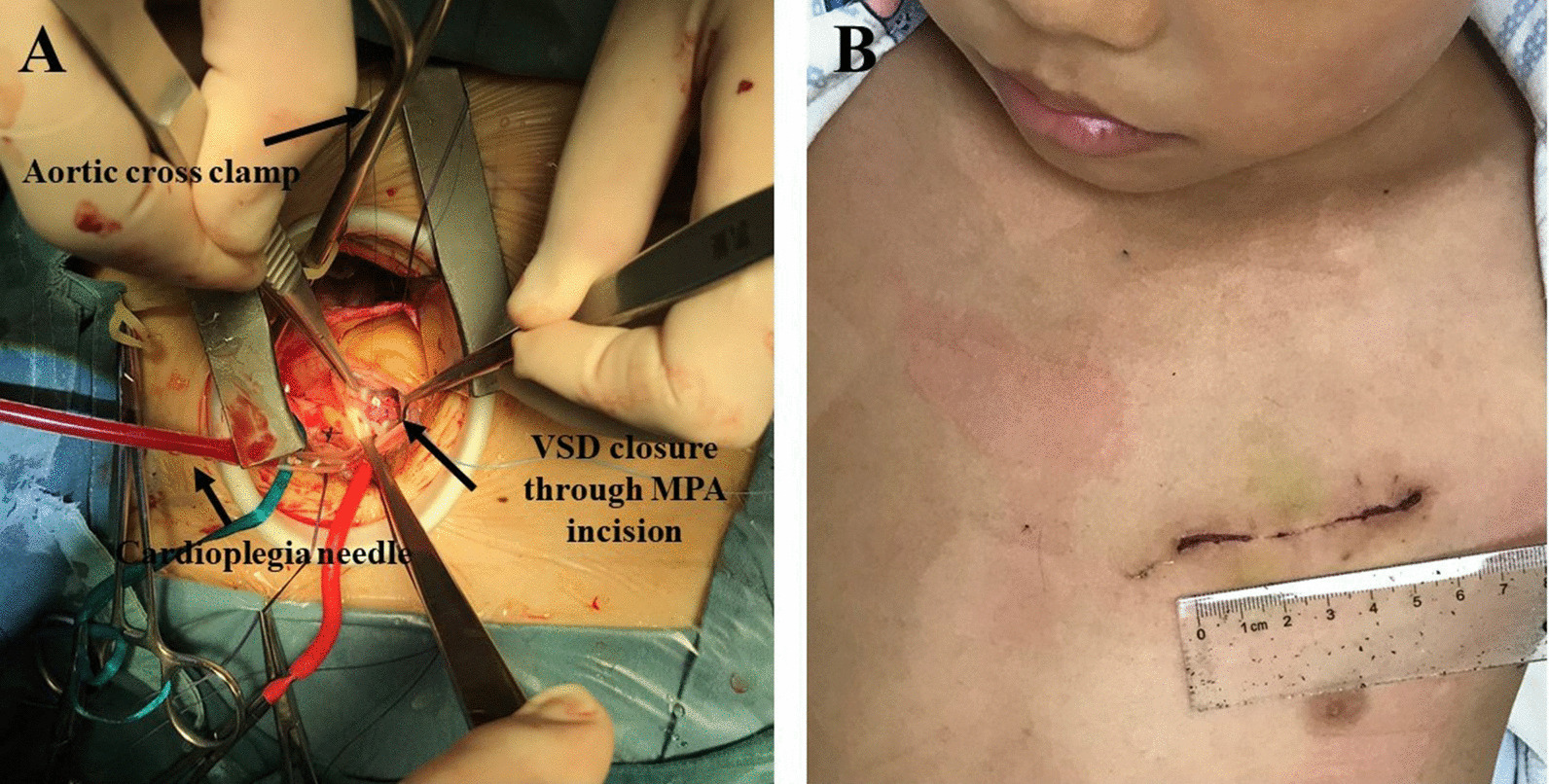
Diagram of a left anterolateral incision to repair subpulmonary ventricular septal defect. **A** A surgeon’s view of the left second intercostal incision, and the defect was repaired through the main pulmonary artery (MPA) incision. **B** Incision before discharge

This approach was indicated in repair of subpulmonary VSD. The patient was placed in the supine position and was intubated using a single-lumen tube. A horizontal incision parallel to the second or the third intercostal space was made. The left internal mammary artery was kept intact as much as possible. Peripheral cardiopulmonary bypass was established by cannulating both the femoral artery and vein. The venous cannula was advanced into the right atrium to obtain better drainage. The aorta was mobilized through the incision; then, a cardioplegia needle was placed at the aortic root. The aorta was cross clamped, and the pulmonary artery was opened approximately 1 cm superior to the pulmonary annulus (Fig. 3). The VSD was exposed and repaired using the same technique from sternotomy. Usually, an SVC cannula was unnecessary because of adequate drainage; however, using an SVC cannula from the incision is also feasible if needed.

### Data collection and statistical analysis

Perioperative and follow-up data were collected. Categorical variables were expressed as frequencies and percentages, and were compared using the chi-square test or Fisher’s exact test between two groups. Continuous variables were presented as means ± standard deviations, as well as ranges. Comparisons between each two groups were performed by Student’s t-test or the Mann–Whitney U test as appropriate. STATA (version 14.0, STATA Corporation, College Station, TX) was used for statistical analysis. Two-tailed *P* values of less than 0.05 were considered statistically significant.

## Results

The preoperative data are summarized in Table 1. This study included 472 patients, among whom 52.1% were male. In terms of the primary diagnosis, among the 335 patients in the RSAVI group, 192 had ASD, 135 had VSD, and 8 had AVSD. Moreover, among the 132 patients in the RALT group, 77 had ASD, 51 had VSD, and 4 had AVSD. The LALT group consisted of five patients, who have subpulmonary VSD. In the RSAVI group, the mean age was 2.4 ± 0.9 years, and the mean weight was 12.4 ± 2.5 kg. Both age and weight were significantly lower than those in the RALT group (age 17.5 ± 4.8 years, weight 54.5 ± 14.9 kg, both *P* < 0.01) and the LALT group (age 15.1 ± 3.8 years, weight 42.1 ± 12.5 kg, both *P* < 0.01).

**Table 1:** Preoperative data

Characteristics	RSAVI group(N = 335)	RALT group(N = 132)	LALT group(N = 5)	*P* value (RALT vs. RSAVI)	*P* value (LALT vs. RSAVI)	*P* value (LALT vs. RALT)
Age (years)	2.4 ± 0.9 (0.8–17)	17.5 ± 4.8* (11–68)	15.1 ± 3.8* (11–20)	< 0.01	< 0.01	0.23
Weight (kg)	12.4 ± 2.5 (7.5–58)	54.8 ± 14.9* (30–83)	42.1 ± 12.5* (30–61)	< 0.01	< 0.01	0.06
Male	172 (51.3%)	71 (53.8%)	3 (60%)	0.63	0.70	0.79
Primary diagnosis						
ASD	192 (57.3%)	77 (58.3%)	0	0.89	0.03	0.02
VSD	135 (40.3%)	51 (38.6%)	5 (100%)			
AVSD	8 (2.4%)	4 (3.0%)	0			

RSAVI, right subaxillary vertical incision; RALT, right anterolateral thoracotomy; LALT, left anterolateral thoracotomy; ASD, atrial septal defect; VSD, ventricular septal defect; AVSD, atrioventricular septal defect

*P < 0.05 when compared to the RSAVI group

All patients underwent MICS uneventfully. There was one transition to a median sternotomy. The patient was a 6-year-old child who underwent ASD repair via RSAVI; however, after the chest was closed, the patient had increased central venous pressure and the hemodynamics was unstable. The decision was to reopen from median sternotomy and fenestrate the ASD patch. This patient recovered well. The intraoperative data and postoperative recovery are summarized in Table 2. Concomitant procedures other than intracardiac defect repair included the relief of right ventricular outflow tract obstruction in nine cases, mitral valve repair in nine cases, and tricuspid valve repair in 37 patients. Three patients required a second pump round of cardiopulmonary bypass to repair residual lesions according to intraoperative transesophageal echocardiography findings (two cases of residual VSDs, and one case of residual mitral regurgitation). The average cardiopulmonary bypass time was 66.6 ± 1.3 min, and the aortic cross clamp time was 37.1 ± 8.9 min.

**Table 2:** Operative data and postoperative recovery

Characteristics	RSAVI group (N = 335)	RALT group (N = 132)	LALT group (N = 5)	*P* value (RALT vs. RSAVI)	*P* value (LALT vs. RSAVI)	*P* value (LALT vs. RALT)
ACC time(min)	35.8 ± 7.5 (11–67)	40.1 ± 10.2* (12–68)	38.8 ± 8.6 (28–49)	< 0.01	0.36	0.80
CPB time(min)	64.9 ± 10.2 (21–105)	71.2 ± 12.3* (33–132)	63.2 ± 10.2 (49–75)	< 0.01	0.70	0.15
Operations						
Isolated ASD repair	187 (55.8%)	60 (45.5%)	0	< 0.01	0.38	0.18
ASD + MVP	1 (0.3%)	2 (1.5%)	0			
ASD + TVP	2 (0.6%)	15 (11.4%)	0			
ASD + RVOTO repair	2 (0.6%)	0	0			
Isolated VSD closure by interrupted stitches	60 (17.9%)	23 (17.4%)	3 (60.0%)			
Isolated VSD patch closure	55 (16.4%)	15 (11.4%)	2 (40.0%)			
VSD repair + MVP	2 (0.6%)	4 (3.0%)	0			
VSD repair + TVP	13 (3.9%)	7 (5.3%)	0			
VSD repair + RVOTO repair	5 (1.5%)	2 (1.5%)	0			
AVSD	8 (2.4%)	4 (3.0%)	0			
Postoperative intubation hours	5.9 ± 2.9 (1–29)	8.6 ± 4.3*(1–42)	3.6 ± 1.9^#^ (2–7)	< 0.01	0.08	0.01
Postoperative ICU days	2.0 ± 1.1 (1–5)	1.9 ± 1.0 (1–6)	2.0 ± 0.7 (1–3)	0.37	1	0.83
Postoperative chest drainage(ml/kg)	7.4 ± 2.1 (3.6–10.3)	6.7 ± 2.1* (3.3–15.5)	6.3 ± 1.8 (3.9–8.5)	< 0.01	0.25	0.68
Postoperative hospitalization days	6.0 ± 1.4 (5–13)	6.5 ± 1.2* (5–19)	6 ± 1.2 (5—8)	< 0.01	0.87	0.27

RSAVI, right subaxillary vertical incision; RALT, right anterolateral thoracotomy; LALT, left anterolateral thoracotomy; ASD, atrial septal defect; MVP, mitral valve repair; TVP, tricuspid valve repair; RVOTO, right ventricular outflow tract obstruction; VSD, ventricular septal defect; AVSD, atrioventricular septal defect

*P < 0.05 when compared to the RSAVI group

^#^*P* < 0.05 when compared to the RALT group

The RSAVI group had slightly shorter bypass time and cross-clamp time than the RALT group. There was no early and late death. Perioperative complications included postoperative chest exploration for bleeding in one patient in the RALT group, and one reoperation due to patch dehiscence 6 days after initial ASD repair in the RSAVI group. Moreover, one patient had temporary neural system dysfunction, and three patients had phrenic nerve paralysis; however, all of them recovered later. Furthermore, 13 patients had pulmonary atelectasis but they recovered before discharge. Most patients were extubated on the operative day (462 patients, 97.9%). The median stay in the intensive care unit was 2 days (range 1–6 days), and the median stay in the hospital was 6 days (range 5–19 days). Echocardiography before discharge indicated five trivial residual intracardiac shunts which healed during the follow-up, and 12 less than mild left or right atrioventricular valve regurgitation. No third-degree atrioventricular block was found, and no patient required reintervention during the follow-up. No new onset of obvious deformity of the chest wall or scoliosis was found. All patients had New York Heart Association I heart function.

## Discussion

Conventional full sternotomy in cardiac surgery has the disadvantages of a long incision, more bleeding and the risk of mediastinitis. Although partial sternotomy shortens the incision, sternal stability is damaged and the patient will require several months to recover back to normal life after surgery. In the last two decades, various minimally invasive techniques were applied in congenital cardiac surgery [14]. Liu et al. from Fuwai Hospital have reported their experience of oblique lateral thoracotomy in 683 children with congenital heart disease [3]. The ages ranged from 4 months to 7 years, and the main diseases in this series were ASD and VSD. A few patients with tetralogy of Fallot were also operated using this technique. However, the length of this incision was not small, and the incision was close to mammary tissue. Bleiziffer et al. have followed 72 female adolescent patients and found that 61% of the patients developed asymmetric breasts in adulthood; therefore, they recommended not using such an incision in adolescent females [15]. To avoid such complications, this group modified their techniques and reported their results in 2005. The modified incision was at the midaxillary level and the length was approximately 5 cm. Thirty-six children with isolated ASD were included in this study, and the outcomes were excellent. The youngest patient in this study was 4 years old [5]. Pretre et al. from Switzerland have reported their experience of using right posterolateral thoracotomy in 80 patients with simple congenital heart disease [4]. In most patients in this group, femoral arterial and venous cannulations were used (87.5%). The mid-term results were good [16]. Palma et al. from Italy have reported excellent outcomes of 132 open-heart surgeries using right anterolateral mini-thoracotomy [17]. Recently, several heart centers from China have reported various modified minimally invasive methods for repairing simple congenital heart diseases. The most commonly used incision was the RSAVI. The incision was modified from an oblique shape to the vertical direction, and its length was also shortened. The indications for such minimally invasive surgeries were expanded, and there was a trend of using a soft tissue retractor instead of a rigid spreader [9, 13, 18, 19]. The safety and efficacy of such strategies were validated, and the outcomes of MICS in children were not inferior to conventional heart surgery.

The indication of MICS and the choice of incision in children are not yet consistent. In the past, the indication was simple ASDs, and the exposure usually was excellent. However, in patients with VSD, surgical exposure was not as good as that in patients with ASD because the VSDs are deep and blocked by tricuspid tissues. With the advancement of instruments and techniques in MICS, the indication was expanded recently. In our opinion, all isolated ASDs of any size or location can be repaired using minimally invasive approaches; however, patients with ASD who have concomitant anomalous pulmonary vein drainage should be evaluated carefully and may not be suitable for this minimally invasive surgery. The most common type of VSD in this study was peri-membranous or inlet muscular VSD because they are usually easily repaired through the tricuspid valve from a right atriotomy. The size of VSD is not crucial for decision making, and both direct suturing and patch closure techniques were used in our patients. In our institution, we prefer to operate on patients whose age is over 8 months and weight is over 8 kg since tissue in infant is fragile. However, Anzhen Hospital recently reported a group of infants less than 5 kg operated via RSAVI, and the outcomes were the same as those operated from median sternotomy [20]. Furthermore, CAVSD repair was reported from lateral mini-thoracotomy in infants as well [9].

An uneventful establishment of cardiopulmonary bypass is fundamental to perform MICS. The diameters of femoral vessels in infants are small, and femoral cannulation may lead to a high incidence of vascular complications; therefore, central cannulation is preferred in such patients. The RSAVI can expose the ascending aorta, SVC and IVC excellently. The arterial cannulation site is usually deep, and aortic cannulation is the most difficult step in most patients. In the literature, some authors have used forceps to grab the tip of the curve arterial cannula which facilitated cannulation [8, 13]; however, mastering this technique is not easy, especially when surgical exposure is limited. In our center, we used a straight arterial cannula with a rigid inner cylinder; and put it inside through an arterial incision. The keys of this step are to open the adventitia within the aortic purse string as much as possible and to rotate the cannula back and forth slightly if we feel resistance. In some older patients with deep thoracic cavities and small incisions, the aorta is punctured using a needle, and a guiding wire is inserted; then an arterial cannula is placed by Seldinger technique. The SVC and IVC cannulas can be either curved or straight, and we prefer using cannulas with thin-walled wire reinforcement, which is flexible to be positioned.

The key point to ensure precise repair is well surgical exposure, especially when the infra-axillary incision is tiny. To obtain better surgical exposure, recently, Heinisch et al. have reported percutaneous cannulation of the IVC in 38 pediatric patients; however, 13.5% of the cases had thrombosis at the cannulation site [21]. In some patients in the RSAVI group, the IVC cannula was placed through the sixth intercostal space, and this puncture site was used to place chest tube at the end of surgery. After cardioplegia was given, the cardioplegia needle and tube were removed, since most simple cardiac defects can be repaired within the protection time of a single dose of cardioplegia. In a few patients with VSD, an ice-cold saline-rinsed gauze was placed behind the heart in the pericardial cavity, which helped push the heart close to the incision; with appropriate retraction, the VSD was exposed well. In case of unrestrictive VSD, patch closure using interrupted stitches was preferred.

We found that the infra-axillary incision was convenient in infants and children; however, it was limited in adolescents and adults since the incision is too far away from the heart. Therefore, we used RALT in such patients. Giordano et al. have reported using similar incision to replace aortic valve in adolescents with bicuspid aortic valve [22]. In this study, the mean age and body weight of the RALT group is significantly higher than those of the RSAVI group. The RALT incision from the fourth intercostal space is close to the heart, and the surgical view is similar to the view from sternotomy. Peripheral cardiopulmonary bypass established by femoral cannulations helps obtain better exposure from the mini-thoracotomy. Recently, the SVC cannula was placed through the right jugular vein, and a Chitwood aortic cross-clamp was used in this study. Furthermore, thoracoscopy assistance and a soft tissue retractor helped us reduce the incision to as short as 4 cm in length. To be noticed, the skin incision should be at the lower margin of the breast tissue in adult females and should be far away from the mammary tissue in prepubescent female children.

The aforementioned two incisions expose ASD and peri-membranous VSD well enough to repair; however, they can hardly expose subpulmonary VSD. A few heart centers have reported their experience of repair doubly committed subarterial VSD using subaxillary incision. VSD was repaired either through the tricuspid valve or through the main pulmonary artery; however, the number of such cases is very limited, and the surgeons are experienced in this field, so the reproducibility is not easy [13, 18]. In this study, only one infant with subpulmonary artery VSD underwent repair using a right subaxillary incision, and the exposure was not good enough for precise repair. Intraoperative transesophageal echocardiography demonstrated residual VSD, and a second cross-clamp was applied to repair the residual defect. After this case, we preferred median sternotomy in infants with subpulmonary artery VSD. Meanwhile, in adolescents and adults with subpulmonary artery VSD, a LALT from the second intercostal space was used. In this study, we reported our preliminary experience in five cases. Femoral arterial and venous cannulations were used. Single IVC cannulation usually achieves adequate drainage, and SVC cannulation is unnecessary in most patients; however, SVC cannulation is possible through the incision if needed after the patient is on bypass. In 2017, authors from China have used this approach to repair subpulmonary artery VSD; however, it was used only in adults, not in children [23]. In 2019, authors from Guangzhou have reported an alternative method using minimal mid-partial sternotomy in 13 patients [24]. Minimally invasive per-ventricular closure of doubly committed subarterial VSD has also been reported, but this technique lacks long-term follow-up results [2]. In our opinion, surgical closure using LALT is preferred in such patient groups. Recently, the LALT incision was also applied to replace the pulmonary valve in seven patients following tetralogy of Fallot repair by Nellis et al. [25].

The lowest weight limit of femoral cannulation is inconsistent. Most surgeons prefer using peripheral cannulation in patients over 30 kg; however, surgeons from Switzerland have dissected the iliac artery in infants with a body weight as low as 10 kg [16]. The reported lowest body surface area using a femoral venous cannulation was approximately 0.3 m^2^, however, the patency of the femoral vein was compromised in 13.5% of the patients [20]. The cutoff body weight is 20 kg in our institution, and preoperative femoral vessels were evaluated using ultrasonography to determine the possibility of femoral cannulation.

This study is limited by its retrospective nature in a single institution, and no comparison was performed between minimally invasive approaches and sternotomy. Pain scores were not evaluated in these patients, and the follow-up time was relatively short. The minimally invasive approaches reported here are unsuitable for all congenital heart diseases, especially in complicated diseases. In infants less than 6 months old, we still prefer median sternotomy due to the fragile heart and lung tissues in infants, whereas a few centers summarized their experience in such patients [19, 20]. In low body weight patients with doubly committed subarterial VSD, we prefer median sternotomy, and finally, if the patient has prior thoracic surgery or has significant adherence in the thoracic cavity, sternotomy is preferred as well.

## Conclusions

In conclusion, MICS is safe and effective in patients with congenital septal defects. RSAVI is suitable in infants and children, while RALT is suitable in adolescents and adults. LALT is feasible to repair subpulmonary VSD in patients over 20 kg. Femoral cannulation is the basis of MICS in adolescents and adults, while jugular venous cannulation and thoracoscopy assistance improve surgical exposure and minimize the length of incision. The application of a soft tissue retractor reduces tissue injury, thus facilitating postoperative recovery. Appropriate preoperative evaluation and surgical strategy achieve good cosmetic and therapeutic effects.

## Data Availability

The dataset generated and/or analyzed during the current study are available from the corresponding author upon reasonable request.

## Abbreviations

MICS: Minimally invasive cardiac surgery
RSAVI: Right sub-axillary vertical incision
RALT: Right anterolateral thoracotomy
LALT: Left anterolateral thoracotomy
ASD: Atrial septal defect
VSD: Ventricular septal defect
AVSD: Atrioventricular septal defect
